# Comprehensive Transcriptomic Analysis of Critical RNA Regulation Associated With Metabolism and Prognosis in Clear Cell Renal Carcinoma

**DOI:** 10.3389/fcell.2021.709490

**Published:** 2021-09-28

**Authors:** Si Liu, Honglan Zhou, Gang Wang, Xin Lian

**Affiliations:** Department of Urology, The First Hospital of Jilin University, Changchun, China

**Keywords:** clear cell renal cell carcinoma, metabolic pathway, prognosis, cell biology 3, urology

## Abstract

This study focuses on investigating the metabolism-related gene profile and prognosis of clear cell renal cell carcinoma (ccRCC) patients. The research data from the Gene Expression Omnibus database, including GSE40435, GSE53757, and GSE53000, were used to analyze the consistently differentially expressed RNAs (cDERs) by the MetaDE limma package. Gene expression profiling associated with metabolism was downloaded from the GSEA database. The cancer genome atlas (TCGA) dataset of ccRCC (the training set) and RNA sequencing data of E-MTAB-3267 from EBI ArrayExpress database (the validation set) were obtained to construct a prognostic model. A series of bioinformatics analysis, including functional enrichment analysis, Cox regression analysis, and constructing a prognostic score (PS) model, was performed. Further *in vitro* experiments including cell proliferation assay and flow cytometry were performed to validate our results. We constructed a metabolism-related prognostic model based on 27 DElncRNAs and 126 DEGs. Gene Set Enrichment Analysis revealed that 19 GO terms and 9 KEGG signaling pathways were significantly associated with lipid metabolic pathways. Furthermore, we generated a nomogram illustrating the association between the identified DERs and the tumor recurrence risk in ccRCC. The results from experimental validation showed that lncRNA SNHG20 was significantly upregulated in tumor tissues compared with adjacent tissues. Knockdown of SNHG20 suppressed the proliferation and induced cell cycle G0/G1 arrest, and apoptosis in ccRCC cells. Our study might contribute to a better understanding of metabolic pathways and to the further development of novel therapeutic approaches for ccRCC.

## Introduction

Clear cell renal cell carcinoma (ccRCC), accounting for approximately 80–90% of renal cell carcinoma cases, is characterized as high metastasis and relapse rate compared with other subtypes (Ljungberg et al., 2019). Essentially, ccRCC is regarded as a metabolic disease with some main risk factors, including diabetes, obesity, and atherosclerosis (Sudarshan et al., 2013). At present, late diagnosis is a major obstacle for improving ccRCC outcomes, with the fact that a portion of patients already have distant metastasis when diagnosed and developed to metastatic recurrence during the follow-up (Rao et al., 2018; Ghatalia et al., 2019). Therefore, there is an urgent need to identify effective prognostic biomarkers/therapeutic targets associated with metabolism for the prediction and treatment of ccRCC (Verbiest et al., 2018).

With the development of gene chips and high-throughput second-generation sequencing technologies, bioinformatics has been widely applied to analyze, and identify genes associated with the progression of renal cell carcinoma. For example, Wan et al. (2020) used the cancer genome atlas (TCGA) database bioinformatics platform to identify DEGs that eliminated patients with high immune and stromal scores in the ccRCC microenvironment. Xu et al. (2019) used available RNA-sequence data from TCGA and Fudan University Shanghai Cancer Center (FUSCC) to reveal that AQP may act as an oncogene and a promising prognostic marker in ccRCC. In addition, Xiao et al. (2020) obtained 293 DEGs by integrating six datasets from the GEO database and CMap analysis. However, the role of the metabolism-related gene set in ccRCC remains largely unclear.

In the present study, we downloaded RNA sequencing and corresponding clinical information from the GEO, TCGA, and EBI ArrayExpress databases. We first identified consistently differentially expressed RNAs (cDERs) by overlapping the candidates through integrated bioinformatics analysis. Subsequently, we selected a cluster of metabolism-related genes to screen for key genes and biological pathways, and construct a prognosis prediction signature. These analyses might provide rational therapeutics in further studies on ccRCC.

## Materials and Methods

### Public Datasets

A total of three datasets (GSE40435, GSE53757, and GSE53000) were obtained by searching the keywords ‘‘Homo sapiens’’ and ‘‘clear cell renal carcinoma’’ and following the criteria (sample size not less than 50 and studies with the presence of normal groups as the control) from National Center for Biotechnology Information (NCBI) GEO^1^ (Clough and Barrett, 2016). A total of 528 ccRCC samples that had both mRNA and lncRNA expression profiling were obtained by mapping the clinical prognosis for each sample from the TCGA data portal,^2^ which were used as training dataset. This expression profiling of the 53 ccRCC samples with corresponding available clinical information about recurrence and prognosis was downloaded from the EBI ArrayExpress^3^ databases, and which were used as a validation dataset.

### Identification of cDERs

The lncRNAs and mRNAs in the mentioned datasets as above were annotated by using Human Genome Organization (HUGO) Gene Nomenclature Committee (HGNC^4^) (Parkinson et al., 2005). To eliminate discrepancies, the MetaDE package Version 1.0.5 in R3.4.1 language (Chang et al., 2013) was used to screen the cDERs, including cDElncRNAs and cDEGs between the ccRCC group, and control group in three datasets (GSE40435, GSE53757, and GSE53000) with the cutoff criteria of false discovery rate (FDR) < 0.05. Next, clustering analysis was performed to detect the distinguishing effect of MetaDE on differential expression in different sample groups.

### Metabolism-Associated DEGs in ccRCC

The genes correlated with three major substances, namely, amino acids and derivatives, carbohydrates, and lipids, were downloaded from the Gene Set Enrichment Analysis database (GSEA^5^) (Subramanian et al., 2005). Metabolism-associated DEGs were obtained by comparing cDEGs in the profiles of GSE40435, GSE53757, and GSE53000.

### Construction of ceRNA Network

The expression levels of metabolism-associated DEGs and cDElncRNAs were obtained in the TCGA data portal, which were used to calculate the pearson correlation coefficient (PCC) using cor. Test function (Zou et al., 2003) in R3.4.1 language. Based on the absolute value of correlation coefficient > 0.4, the regulation network of metabolism-associated DEGs and cDElncRNAs was plotted by Cytoscape (3.6.1) (Shannon et al., 2003). Subsequently, DAVID online tool version 6.8 (Huang da et al., 2009) was used to perform gene ontology (GO) biological process annotation and kyoto encyclopedia of genes and genomes (KEGG) pathway analyses for DEGs in the co-expression network. All significant GO or KEGG terms based on the threshold of *p* < 0.05 should be composed of at least two DEGs.

### Establishment of the Prognostic Gene Signature

Univariate Cox proportional hazard regression analysis in Survival package (Version2.41–1^6^) (Wang et al., 2016) was conducted to screen the metabolism-associated DEGs and cDElncRNAs in the ceRNA network significantly associated with the prognosis of TCGA-ccRCC cohorts with the threshold of log-rank *p*-value < 0.05. Then, a multivariate Cox regression analysis was conducted to assess the contribution of metabolism-associated DEGs and cDElncRNAs as independent prognostic factors for patient survival. Next, we applied a L1 penalized lasso estimation-based Cox-proportional hazards (Cox-PH) model (Tibshirani, 1997) in the penalized package (Version 0.9–50^7^) of R3.4.1 (Goeman, 2010) to further screen the optimum prognostic signature cDERs. The optimal lambda was determined according to the maximal cross-validation likelihood run 1,000 times. Subsequently, a linear combination method was adopted to assemble expression level and coefficient of each gene to get a risk score formula: Prognostic score (PS) = Σβ_DERs_ × Exp_DERs_. Here, β_DERs_ represent the LASSO coefficients of signature cDERs, and Exp_DERs_ represent the expression levels of signature cDERs. The patients in the training dataset were stratified into high-risk (PS > median value) and low-risk (PS < median value) groups based on the median risk score as the cutoff. The Kaplan–Meier survival analysis with log-rank test were also used to compare the survival difference between the above two groups. The time-dependent receiver operating characteristic (ROC) curve was drawn to evaluate the predictive power of this signature. Similarly, the predictive value of the prognostic gene signature was further investigated in the validation dataset.

### Identification of Independent Prognostic Parameters of ccRCC

To identify independent prognostic parameters and to validate the independent prognostic value of the gene signature, univariate Cox regression analysis was first performed in the TCGA dataset on the prognostic gene signature and clinicopathological parameters including age, gender, pathologic M, N, T, and PS model status. These parameters with *p* < 0.05 based on the univariate analysis were further included in the multivariate Cox regression analysis. To further investigate the relationship between independent prognostic factors and risk grouping, we performed risk stratification analysis based on screened prognostic factors in the TCGA dataset for studying the prognosis of target clinical factors in high-risk and low-risk groups.

### Building and Validating a Predictive Nomogram

Nomograms are widely applied to predict cancer patients’ prognoses. Here, a composite nomogram was constructed using R3.4.1 rms package Version 5.1-2^8^ (Anderson et al., 1989; Eng et al., 2015) based on all independent prognostic parameters screened by the multivariable Cox regression analysis to assess the probability of 3– and 5-year overall survival for ccRCC patients. Subsequently, we constructed the clinical prognosis models based on clinicopathological parameters, signature lncRNAs, or mRNAs in PS model, respectively, which were compared with the PS prognostic model by drawing ROC curves with two parameters (C-index and AUROC).

### Clinical Sample Preparation

A total of 50 paired ccRCC tissue and adjacent normal tissue samples were collected from ccRCC patients who underwent radical nephrectomy at the First Hospital of Jilin University. The patients whose tissues were used in the present study had never received chemotherapy or radiotherapy. All the samples were stored at −80°C until use. The study protocol was approved by the ethics committee of the First Hospital of Jilin University.

### Cell Transfection

Two ccRCC cell lines (786-O and ACHN) were purchased from the American Type Culture Collection (Manassas, VA, United States) and cultured in Dulbecco’s modified Eagle’s medium (Thermo Fisher Scientific, Inc., Waltham, MA, United States) with 10% FBS at 37°C in a 5% CO_2_ incubator. For transient transfection, cells were plated into six-well plates (2 × 10^5^/well) and transfected with small interfering RNA targeting SNHG20 (si-SNHG20#1, 2, and 3) or negative control (si-NC) synthesized by GenePharma (Shanghai, China) using Lipofectamine 2,000 (Invitrogen, CA, United States).

### Quantitative Real-Time PCR

Total RNA was extracted from tissue samples or cell lines using TRIzol reagent (Thermo Fisher Scientific) and reverse transcribed into cDNA using a First Strand cDNA Synthesis Kit (Takara Biotechnology Co., Ltd., Dalian, China) according to the manufacturer’s instructions. Quantitative real-time PCR was carried out using SYBR Premix ExTaq^TM^ (TaKaRa, Dalian, China). Relative expression levels of mRNAs and lncRNAs were calculated using the 2^–ΔΔ^ ^Ct^ method with GAPDH as the internal control.

### Cell Counting Kit-8 Assay

The transfected cells were seeded into 96-well plates at a density of 3,000 cells per well. Then, 10 μl of CCK-8 reagents (Beyotime Institute of Biotechnology, Shanghai, China) was added into each well at 0, 24, 48, and 72 h. After incubation for 2 h at 37°C, the absorbance at each time point was measured at a wavelength of 450 nm by a microplate reader.

### Colony Formation Assay

The transfected cells at a density of 300 cells per well were seeded into six-well plates and cultured for 14 days. The colonies (>50 cells per colony) were fixed with 4% paraformaldehyde for 15 min and stained with 0.5% crystal violet for 10 min. Subsequently, the number of colonies was counted from three randomly chosen fields under an Olympus microscope (Tokyo, Japan).

### Flow Cytometry Analysis

After 48 h transfection, cells were harvested for the flow cytometry detection. Cell cycle distribution was analyzed using a CycleTESTTM PLUS DNA Reagent Kit (BD Biosciences) according to the standard protocol. Briefly, cells were stained with propidium iodide (PI), mixed with RNase A at 37°C for 30 min, and then analyzed by a flow cytometer (BD Biosciences). Apoptotic assay was performed with an Annexin V–FITC Apoptosis Detection Kit, which was detected by flow cytometry.

### Statistical Analysis

The measurement data were expressed by the mean ± standard deviation (SD). Prism 6.0 software (GraphPad, Inc., La Jolla, CA, United States) was used for statistical analysis. The differences between the tumor tissue and normal colonic mucosa were analyzed by a paired *t*-test. The comparison between two groups was estimated by Student’s *t*-test and comparison among more than three groups was analyzed *via* one-way analysis of variance (ANOVA) followed by Dunnett’s test. All values of *p* less than 0.05 were considered statistically significant.

## Results

### Screening of Candidate Metabolism-Associated cDEGs

After annotation ensemble ID, we obtained 297 lncRNAs and 15,840 mRNAs in the datasets as above. Using the threshold of FDR < 0.05 in MetaDE, a total of 2115 cDERs (cDEGs and cDElncRNAs), including 785 downregulated, and 1,330 upregulated genes were identified between ccRCC and normal samples in GSE40435, GSE53757, and GSE53000. The results from clustering analysis not only could distinguish ccRCC samples from normal samples, but also present the consistency of the 2115 cDEGs in each dataset. Then, we downloaded metabolism-associated genes, including 372 amino acid and derivative metabolism-associated genes, 293 carbohydrate metabolism-associated genes, and 738 lipid metabolism-associated genes. Following the intersection of cDEGs, we obtained 139 candidate metabolism-associated cDEGs, as described by a Venn diagram.

### ceRNA Network in ccRCC

**FIGURE 1:**
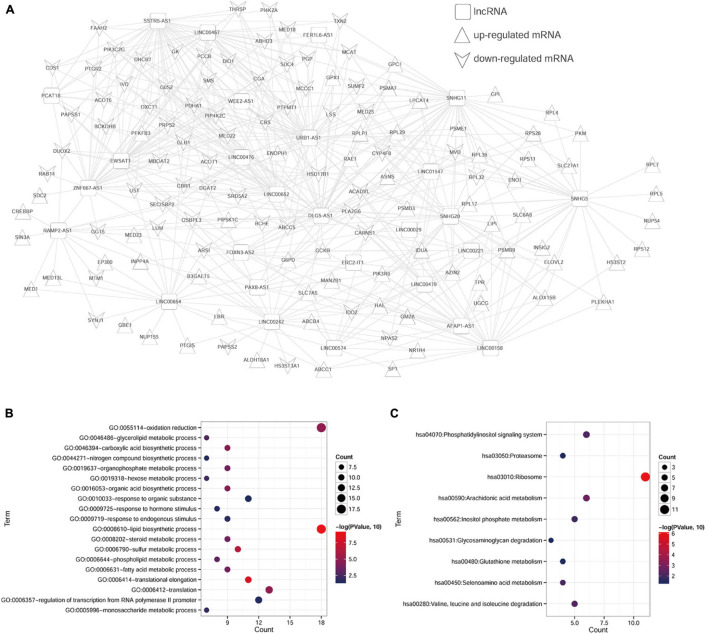
Enrichment analyses of genes in ceRNA network. **(A)** The lncRNA–mRNA ceRNA network in ccRCC. The squares represent lncRNAs, the equilateral triangles represent upregulated mRNAs, and inverted triangles represent downregulated mRNAs. **(B)** GO-BP pathway maps related to mRNAs in the ceRNA network. **(C)** KEGG pathway maps associated with mRNAs in the ceRNA network.

After calculating the PCC between 139 cDEGs and cDElncRNAs in the TCGA database, the pairs with a PCC value of more than 0.4 were selected to determine the final ceRNA relationship for a total of 521 pairs, and 153 nodes. As shown in Figure 1A, we performed and visualized the lncRNA–mRNA network using Cytoscape. To further clarify the potential biological functions of mRNAs in the ceRNA network, the DAVID online tool was used to perform functional enrichment analysis. From the 124 target mRNAs, a total of 19 GO biological process categories and 9 KEGG pathways were enriched with a cutoff criterion of *p* < 0.05 (Table 1). In particular, these mRNAs are primarily associated with lipid biosynthetic process, translational elongation, and sulfur metabolic process, as well as participated in ribosome, arachidonic acid metabolism, and seleno-amino acid metabolism. The visualization results are shown in Figures 1B,C.

**TABLE 1:** Functional enrichment analysis of mRNAs in ceRNA network.

Category	Term	Count	*P*-value
Biology process	GO:0008610∼lipid biosynthetic process	18	6.75E-10
	GO:0006414∼translational elongation	11	7.23E-09
	GO:0006790∼sulfur metabolic process	10	3.27E-07
	GO:0055114∼oxidation reduction	18	1.16E-05
	GO:0006412∼translation	13	1.28E-05
	GO:0046394∼carboxylic acid biosynthetic process	9	3.31E-05
	GO:0016053∼organic acid biosynthetic process	9	3.31E-05
	GO:0006631∼fatty acid metabolic process	9	1.84E-04
	GO:0019637∼organophosphate metabolic process	9	1.97E-04
	GO:0008202∼steroid metabolic process	9	2.11E-04
	GO:0006644∼phospholipid metabolic process	8	8.15E-04
	GO:0046486∼glycerolipid metabolic process	7	1.88E-03
	GO:0019318∼hexose metabolic process	7	4.37E-03
	GO:0005996∼monosaccharide metabolic process	7	8.72E-03
	GO:0009719∼response to endogenous stimulus	9	1.57E-02
	GO:0009725∼response to hormone stimulus	8	2.76E-02
	GO:0006357∼regulation of transcription from RNA polymerase II promoter	12	3.04E-02
	GO:0044271∼nitrogen compound biosynthetic process	7	4.58E-02
	GO:0010033∼response to organic substance	11	6.20E-02
KEGG Pathway	hsa03010: Ribosome	11	8.86E-07
	hsa00590: Arachidonic acid metabolism	6	1.83E-03
	hsa00280: Valine, leucine and isoleucine degradation	5	5.00E-03
	hsa04070: Phosphatidylinositol signaling system	6	6.18E-03
	hsa00450: Selenoamino acid metabolism	4	7.78E-03
	hsa00562: Inositol phosphate metabolism	5	1.03E-02
	hsa03050: Proteasome	4	3.82E-02
	hsa00531: Glycosaminoglycan degradation	3	4.33E-02
	hsa00480: Glutathione metabolism	4	4.47E-02

### Construction and Evaluation of Prognosis Prediction Model

**FIGURE 2:**
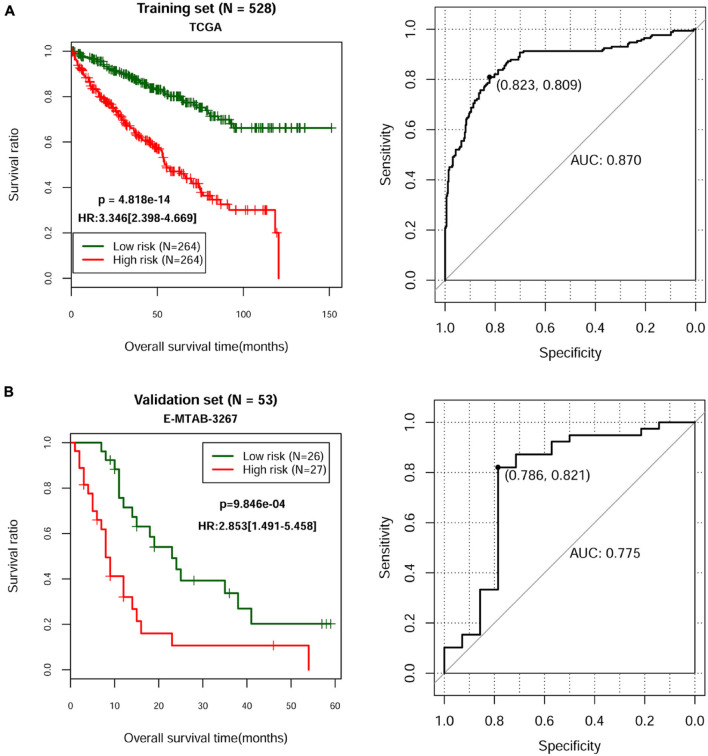
Kaplan–Meier and time-dependent ROC curves for the prognostic model in the TCGA ccRCC cohort **(A)** and in the E-MTAB-3267 ccRCC cohort **(B)**. The Kaplan–Meier survival curves **(left panel)** show the overall survival based on the relatively high- and low-risk patients divided by the optimal cutoff point. Time-dependent ROC curve analysis **(right panel)** of survival prediction by the prognostic model.

According to the univariate Cox regression analysis, a total of 83 cDERs in the ceRNA network, namely, 67 mRNAs and 16 lncRNAs, and significantly related to overall survival in ccRCC patients when the log-rank *p*-value < 0.05. After multivariate Cox regression analysis, we obtained 16 independent prognostic-related cDERs, namely, 14 mRNAs, and 2 lncRNAs. Then, a L1 penalized estimation-based Cox-PH model was performed in the training dataset to further narrow down the 16 independent prognostic-related cDERs. A total of 13 genes were identified and subsequently used to construct a prognostic gene signature (Table 2). We then calculated the 13-gene-based PS for each patient in the training dataset and used the median value of PS as the cutoff point. A total of 264 patients were classified as high risk because their PS was greater than the cutoff value, while the other 264 patients were assigned to the low-risk group, and with PS below the cutoff point. Kaplan–Meier curve and time-dependent ROC were used to assess the prognostic capacity of the 13-gene signature. As shown in Figure 2A, patients at low risk survived significantly longer than those at high risk (HR = 3.346, 95% CI = 2.398–4.669, *p* = 4.818E-14, and AUC = 0.870). Consistent results were also observed in the validation dataset (HR = 2.853, 95% CI = 1.491–5.458, *p* = 9.846E-04, and AUC = 0.775) (Figure 2B). Collectively, our results indicated a good performance of the 13-gene signature for survival prediction.

**TABLE 2:** The optimum prognostic signature DERs.

Symbol	Type	Multi-variate Cox regression analysis			LASSO coefficient
		HR	95% CI	*P*-value	
ABCB4	mRNA	0.7892	0.6814–0.9141	7.95E-04	–0.14612528
ASNS	mRNA	1.3709	1.1673–1.9431	3.81E-02	0.30345902
CREBBP	mRNA	0.3805	0.1214–0.921	4.86E-02	–0.30492645
FAAH2	mRNA	0.7475	0.5891–0.9485	8.30E-03	–0.21756381
HS3ST2	mRNA	0.901	0.8037–0.998	3.69E-02	–0.11888964
HS3ST3A1	mRNA	1.1165	1.0009–1.2454	2.41E-02	0.09363597
MED25	mRNA	2.2648	1.0533–4.8699	1.82E-02	0.13118045
OXCT1	mRNA	0.7556	0.561–0.9178	3.26E-02	–0.11694011
PIK3R6	mRNA	1.3622	1.1207–1.6557	9.55E-04	0.25617027
PLA2G6	mRNA	1.5782	1.0939–2.2769	7.35E-03	0.05863893
RAB14	mRNA	2.5939	1.8899–7.5604	4.04E-02	0.06216072
SNHG11	lncRNA	0.5328	0.2724–0.8422	3.30E-02	–0.02172556
SNHG20	lncRNA	1.5678	1.1605–2.5591	3.60E-02	0.02632259

### Evaluation of Prognostic Factors in ccRCC

**FIGURE 3:**
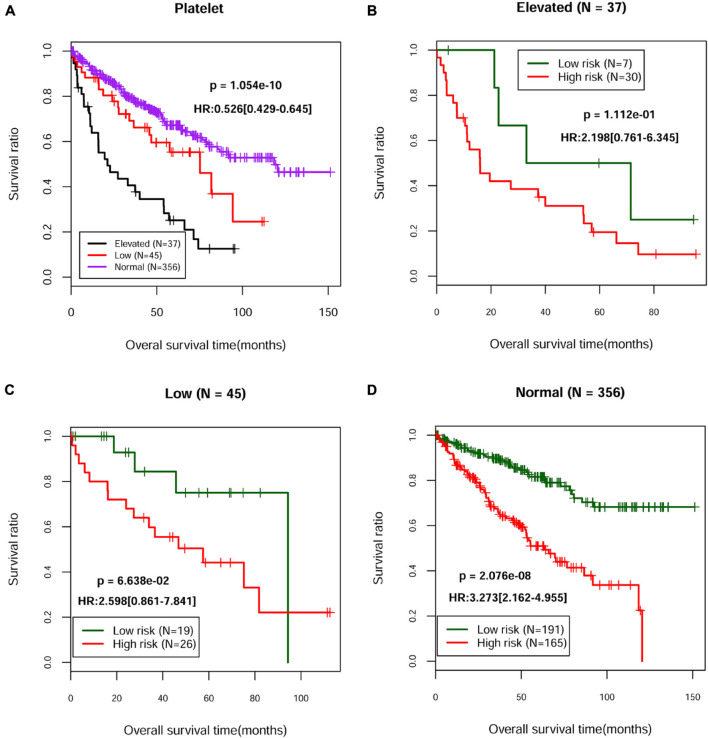
The Kaplan–Meier curves of independent prognostic clinical factors. **(A)** Kaplan–Meier curves of the survival of patients with different platelet status in the TCGA dataset. Kaplan–Meier curves of the survival of patients from the high-risk group and the low-risk group in elevated **(B)**, low **(C)**, or normal **(D)** platelet status, respectively.

Based on the complete clinical information provided by the TCGA ccRCC dataset (*n* = 528), we performed univariate and multivariate Cox regression analyses to identify the prognostic factors of overall survival for ccRCC. As illustrated in Table 3, univariate analysis revealed that age (*p* = 8.493E-06), pathologic M (*p* = 1.110E-16), pathologic N (*p* = 5.359E-05), pathologic T (*p* = 1.221E-15), pathologic stage (*p* = 2.000E-16), neoplasm histologic grade (*p* = 7.772E-16), hemoglobin result (*p* = 2.739E-07), platelet qualitative (*p* = 1.054E-10), and PS model status (*p* = 4.818E-14) were significantly correlated with overall survival of ccRCC. Multivariate analysis further confirmed that platelet qualitative (*p* = 2.478E-03) and PS model status (*p* = 2.610E-04) were independent risk factors of overall survival. Kaplan–Meier curve showed that patients at normal platelet status had better survival prognosis, compared with those at elevated or low platelet status (Figure 3A). Subsequently, we performed risk stratification analysis to analyze the correlation between the high-/low-risk group and survival prognosis in patients with different platelet qualitative samples. The results showed that patients at elevated (Figure 3B), low (Figure 3C), or normal (Figure 3D) platelet status in the low-risk group had obviously longer survival time compared with corresponding patients in the high-risk group.

**TABLE 3:** The independent prognostic clinical factors according to univariate and multivariate Cox regression analyses.

Clinical characteristics	TCGA (*N* = 528)	Uni-variable Cox		Multi-variable Cox	
		HR (95% CI)	*P*-value	HR (95% CI)	*P*-value
Age (years, mean ± SD)	60.53 ± 12.14	1.029 [1.016–1.042]	8.493E-06	1.019 [0.998–1.040]	7.797E-02
Gender (Male/Female)	344/184	0.950 [0.697–1.295]	7.456E-01	–	–
Pathologic M (M0/M1/-)	420/78/30	4.320 [3.161–5.904]	1.110E-16	2.687 [0.944–7.642]	6.392E-02
Pathologic N (N0/N1/-)	239/16/273	3.414 [1.812–6.434]	5.359E-05	0.569 [0.207–1.564]	2.745E-01
Pathologic T (T1/T2/T3/T4)	269/69/179/11	1.918 [1.628–2.260]	1.221E-15	1.179 [0.602–2.309]	6.306E-01
Pathologic stage (I/II/III/IV/-)	263/57/123/83/2	1.884 [1.652–2.150]	2.000E-16	1.058 [0.524–2.134]	8.757E-01
Neoplasm histologic grade (G1/G2/G3/G4/-)	13/227/205/75/8	2.304 [1.880–2.824]	7.772E-16	1.198 [0.822–1.746]	3.476E-01
Hemoglobin result (Elevated/Low/Normal/-)	5/260/183/80	0.428 [0.306–0.560]	2.739E-07	0.940 [0.544–1.6247]	8.252E-01
Platelet qualitative (Elevated/Low/Normal/-)	37/45/356/90	0.526 [0.429–0.645]	1.054E-10	0.611 [0.444–0.841]	2.478E-03
Serum calcium (Elevated/Low/Normal/-)	10/203/149/166	0.949 [0.686–1.312]	7.517E-01	–	–
White cell count (Elevated/Low/Normal/-)	162/8/265/93	1.139 [0.958–1.355]	1.353E-01	–	–
PS model status (High/Low)	264/264	3.346 [2.398–4.669]	4.818E-14	2.807 [1.613–4.885]	2.610E-04
Death (Dead/Alive)	173/355	–	–	–	–
Overall survival time (months, mean ± SD)	45.03 ± 32.80	–	–	–	–

*HR, hazard ratio; CI, confidence interval; SD standard deviation.*

### Building and Validating a Predictive Nomogram

**FIGURE 4:**
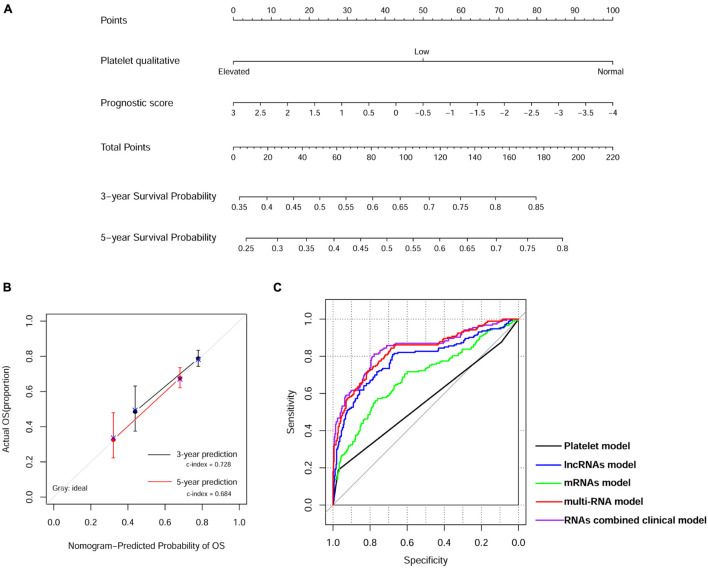
Nomogram predicting overall survival for ccRCC patients. **(A)** Nomogram survival prediction model consists of platelet qualitative and prognostic score based on the 13-lncRNA signature. **(B)** The calibration plot for internal validation of the nomogram. The *y*-axis represents actual survival, and the *x*-axis represents nomogram-predicted survival. **(C)** Time-dependent ROC curve analysis of survival prediction by different prognostic models.

We then built a nomogram to predict the 3-year and 5-year survival probability of ccRCC patients using two independent prognostic factors including platelet qualitative and PS model status. As shown in Figure 4A, each factor was assigned points in proportion to its risk contribution to survival. Calibration curves indicated that actual and predicted survival matched very well (Figure 4B). Next, we constructed different prognostic models, including platelet, lncRNA, mRNA, multi-RNA, and RNA combined clinical models, and drew ROC curves to evaluate their predictive power (Figure 4C). We found that the RNA combined clinical model presented higher AUROC and C-index values (Table 4), which may be the best model in predicting overall survival.

**TABLE 4:** Comparison of the different models.

Model	AUROC	C-index	*P*-value	Specificity	Sensitivity
Platelet model	0.557	0.592	6.304E-06	0.572	0.588
lncRNAs model	0.691	0.589	1.733E-04	0.761	0.572
mRNAs model	0.784	0.719	0	0.679	0.809
multi-RNA model	0.830	0.726	0	0.811	0.723
RNAs combined clinical model	0.840	0.740	0	0.778	0.812

### Knockdown of SNHG20 Suppressed the Cell Proliferation of ccRCC Cells

**FIGURE 5:**
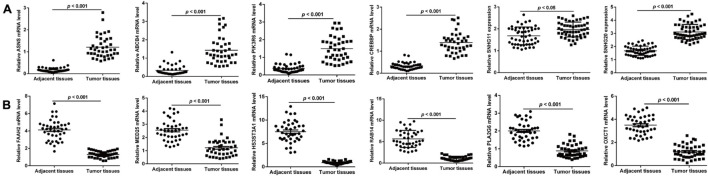
Validation on the screened prognostic signature DERs, including six upregulated DERs **(A)** and six downregulated DERs **(B)** in paired samples of ccRCC and adjacent tissues.

**FIGURE 6:**
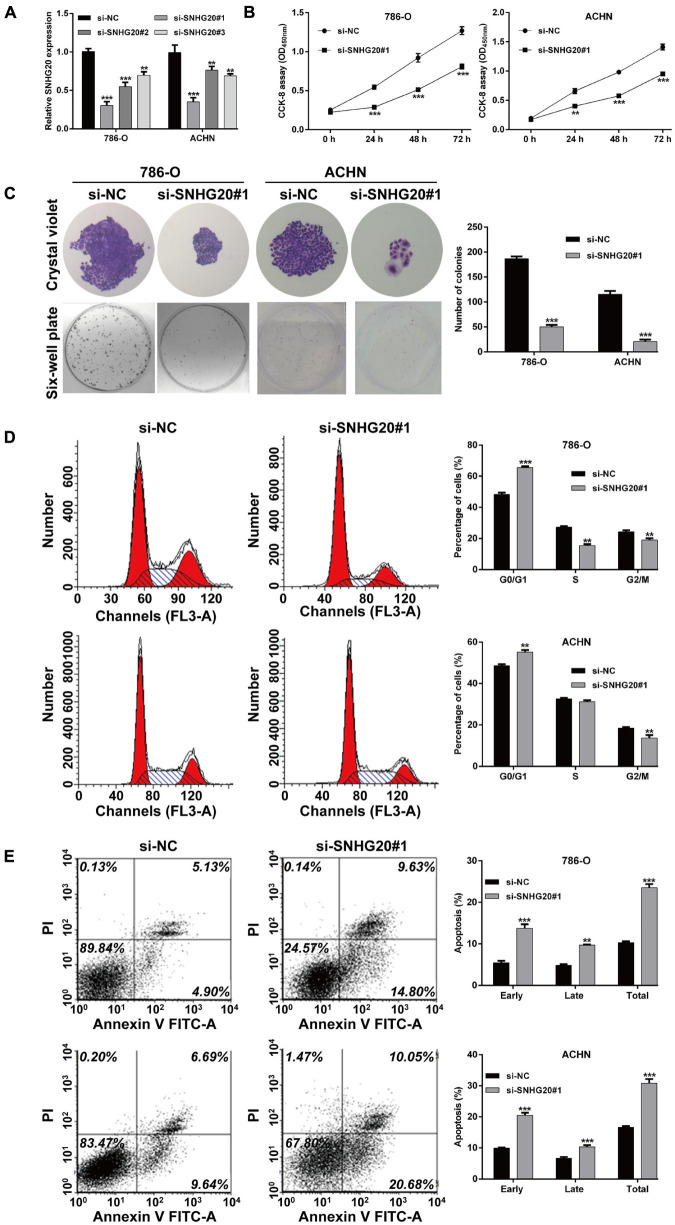
SNHG20 silencing inhibited cell proliferation and cell cycle progression and promoted cell apoptosis in ccRCC cells. 786-O and ACHN cells were transfected with si-SNHG20#1, 2, 3, or si-NC. **(A)** The level of SNHG20 in transfected cells was measured by quantitative real-time PCR. **(B)** The cell viability in transfected cells was assessed *via* CCK-8 assay. **(C)** Colony formation assays performed with the 786-O and ACHN cells transfected with si-SNHG#1 or si-NC. **(D)** Cell cycle distribution and **(E)** apoptotic status were analyzed in transfected 786-O and ACHN cells using flow cytometry analysis. Statistical significance of differences is indicated as follows: **p < 0.01 and ***p < 0.001.

Next, we validated the 13 screened prognostic signature DERs, namely, 11 mRNAs and 2 lncRNAs, and using quantitative real-time PCR analysis. Consistent with the bioinformatics analysis, the expression levels of ABCB4, ASNS, CREBBP, PIK3R6, SNHG11, and SNHG20 were upregulated (Figure 5A), while the expression levels of FAAH2, HS3ST3A1, MED25, OXCT1, PLA2G6, and RAB14 were downregulated (Figure 5B) in ccRCC tissues compared with adjacent normal tissue samples. Next, we selected two lncRNAs, namely, SNHG11 and SNHG20, and in the subsequent analysis. Considering SNHG20 presented stronger upregulation compared with SNHG11, we selected SNHG20 for further *in vitro* experiments by performing loss-of-function assays. We first designed three silent interference sequences for SNHG20. Then, the silent interference efficiency was detected by the PCR, which showed that the si-SNHG20#1 sequence exhibited the highest silent interference efficiency (all *p* < 0.05, Figure 6A). Therefore, the si-SNHG20#1 sequence was selected for subsequent experiments. The results from the CCK-8 assay (Figure 6B) and the colony formation assay (Figure 6C) consistently demonstrated that knockdown of SNHG20 significantly impaired cell viability and proliferation ability in 786-O, and ACHN cells. Subsequently, flow cytometry assays were performed to evaluate the impact of SNHG20 on the ccRCC cell cycle and apoptosis. As shown in Figure 6D, the percentage of cells at G0/G1 phase was significantly increased, while cells at S phase, and G2/M phase was decreased in 786-O and ACHN cells. In addition, we observed that knockdown of SNHG20 markedly promoted the percentage of apoptotic cells in 786-O and ACHN cells (Figure 6E).

## Discussion

To our best knowledge, abnormality of cancer metabolic processes, such as glucose metabolism and amino acid metabolism, and is the hallmark of cancer (Kroemer and Pouyssegur, 2008). The limitation of most previous studies is that they focused on a single genetic event or the results from a single cohort study. This study used bioinformatics to screen metabolism and prognosis-related genes based on the data of ccRCC gene expression in the GEO and TCGA databases. A total of 139 candidate metabolism-associated cDEGs were obtained and used to construct the ceRNA network in ccRCC, including 27 lncRNAs, and 124 mRNAs. We performed functional enrichment analysis and found that these 124 mRNAs were enriched in metabolism-related GO terms and KEGG pathways, including lipid biosynthetic process, translational elongation, and sulfur metabolic process, as well as ribosome, arachidonic acid metabolism, and seleno-amino acid metabolism pathways. Previous studies have shown that kidney cancer is a disease of dysregulated cellular metabolism (Cairns et al., 2011). On this basis, the lncRNA–mRNA co-expression network was performed and 153 co-expression modules, namely, and 27 lncRNAs and 126 mRNAs, were identified. For instance, Acyl-CoA thioesterase 1 (ACOT1), as an important enzyme in fatty acid metabolism, catalyzes the reaction of fatty acyl-CoAs to CoA-SH and free fatty acids (Dongol et al., 2007). A recent study by Fang et al. (2018) showed that there is abnormal metabolism of lipids and fatty acids during gastrointestinal tumor metabolism. Phosphoinositide-3-kinase regulatory subunit 6 (PIK3R6) has been shown to be upregulated in the diabetes rat model, which was associated with the development of type II diabetes in mice (Zhang et al., 2017). Moreover, PIK3R6 has been demonstrated to promote the proliferative and migratory potentials in ovarian cancer cells (Liu et al., 2020). These reports were consistent with our data that downregulated ACTO1 and upregulated PIK3R6, and were correlated with dysregulated cellular metabolism in ccRCC.

Next, we constructed a 13-core prognostic gene signature that can be used to stratify patients into a high-risk group and a low-risk group. We further evaluated the prognostic value of the key genes with ROC and KM survival analysis in the validation dataset. More importantly, we screened two independent risk factors, namely, platelet qualitative (*p* = 2.478E-03), and PS model status (*p* = 2.610E-04). We further confirmed that platelet qualitative and PS model status can availably predict the overall survival for 3 and 5 years in patients with ccRCC. Of these 13 core prognostic gene signatures, ABCB4 (Olsen et al., 2020) and CREBBP (Jin et al., 2018) were involved in lipid metabolism. FAAH2 (Sirrs et al., 2015) was involved in endocannabinoid metabolites. HS3ST2 was involved in energy metabolism (Zhu N. et al., 2020). Consistent with our prediction, quantitative real-time PCR analysis further validated the expression levels of the 13-prognostic gene signature.

Notably, SNHG11 and SNHG20, as the only two lncRNAs included in this 13-prognostic gene signature, were selected for further validation. Our data showed that the expression levels of SNHG11 and SNHG20 were significantly elevated in ccRCC tissue compared with normal tissue. Subsequently, we explored the functional role of SNHG20 in ccRCC *in vitro*. The results revealed that knockdown of SNHG20 significantly suppressed cell proliferation and induced cell cycle G0/G1 arrest and apoptosis in 786-O, and ACHN cells. In agreement with our data, SNHG20 expression markedly increased in tumor tissues and promoted the malignant progression in laryngeal squamous cell carcinoma (Li et al., 2019), epithelial ovarian cancer (Wang et al., 2019), and osteosarcoma (Zhang et al., 2018). Upregulated SNHG20 expression is capable of serving as an innovative predictive factor of inferior clinical outcomes in cancer patients (Zhu H. et al., 2020). On the other hand, one limitation of our study is that our research regarding the 13 genes that make up our signature and predict survival is insufficient; further validation in clinical practice is needed.

## Conclusion

We identified a 13-gene risk signature related to metabolism to independently predict the prognosis of ccRCC patients and validated the functional role of SNHG20 in ccRCC cells. Our findings could provide novel metabolism-related targets for studies of the pathogenesis of ccRCC.

## Data Availability Statement

The datasets presented in this study can be found in online repositories. The names of the repository/repositories and accession number(s) can be found in the article/supplementary material.

## Ethics Statement

The clinical sample preparation protocol was reviewed and approved by the Ethics Committee of the First Hospital of Jilin University.

## Author Contributions

SL and XL contributed to the study conception and design. All authors collected the data and performed the data analysis, contributed to the interpretation of the data and the completion of the figures and tables, and contributed to the drafting of the article and final approval of the submitted version.

## Conflict of Interest

The authors declare that the research was conducted in the absence of any commercial or financial relationships that could be construed as a potential conflict of interest.

## Publisher’s Note

All claims expressed in this article are solely those of the authors and do not necessarily represent those of their affiliated organizations, or those of the publisher, the editors and the reviewers. Any product that may be evaluated in this article, or claim that may be made by its manufacturer, is not guaranteed or endorsed by the publisher.

## Footnotes

1
https://www.ncbi.nlm.nih.gov/geo/

2
https://tcga-data.nci.nih.gov/tcga/

3
https://www.ebi.ac.uk/arrayexpress/

4
http://www.genenames.org/

5
http://software.broadinstitute.org/gsea/downloads.jsp

6
http://bioconductor.org/packages/survivalr/

7
http://bioconductor.org/packages/penalized/

8
https://cran.r-project.org/web/packages/rms/index.html

## Abbreviations

ccRCC: clear cell renal cell carcinoma
TCGA: the cancer genome atlas
cDERs: consistently differentially expressed RNAs
PS: prognostic score
FUSCC: Fudan University Shanghai Cancer Center
HGNC: Human Genome Organization Gene Nomenclature Committee
PCC: pearson correlation coefficient
GO: gene ontology
KEGG: kyoto encyclopedia of genes and genomes
ROC: receiver operating characteristic
CCK-8: cell counting kit-8
Cox-PH: L1 penalized lasso estimation-based Cox-proportional hazards.
