# Corrigendum: Comprehensive transcriptomic analysis of critical RNA regulation associated with metabolism and prognosis in clear cell renal carcinoma

**DOI:** 10.3389/fcell.2023.1216783

**Published:** 2023-06-01

**Authors:** Si Liu, Honglan Zhou, Gang Wang, Xin Lian

**Affiliations:** Department of Urology, The First Hospital of Jilin University, Changchun, China

**Keywords:** clear cell renal cell carcinoma, metabolic pathway, prognosis, cell biology 3, urology

In the published article, there was an error in the Funding statement. Some numbers were erroneously omitted from the funding number. (No. YDZJ2021ZYTS110) was published instead of (No. YDZJ202101ZYTS110). The correct Funding statement appears below.

“This work was supported by the Natural Science Foundation of Jilin Province (No. YDZJ202101ZYTS110).”

The authors apologize for this error and state that this does not change the scientific conclusions of the article in any way. The original article has been updated.

