# Corrigendum: Comprehensive transcriptomic analysis of critical RNA regulation associated with metabolism and prognosis in clear cell renal carcinoma

**DOI:** 10.3389/fcell.2024.1396267

**Published:** 2024-04-03

**Authors:** Si Liu, Honglan Zhou, Gang Wang, Xin Lian

**Affiliations:** Department of Urology, The First Hospital of Jilin University, Changchun, China

**Keywords:** clear cell renal cell carcinoma, metabolic pathway, prognosis, cell biology 3, urology

In the published article, there was an error in Figure 6C (right down panel) which was presented for ACHN cells as published. Since it is a bioinformatics paper, we reviewed multiple papers for the analysis in the paper. During the assembly of the figures, an incorrect copy of the colony formation photo was applied for ACHN cells. The corrected Figure 6C (right down panel) which was presented for ACHN cells and its caption appears below.

**FIGURE 6:**
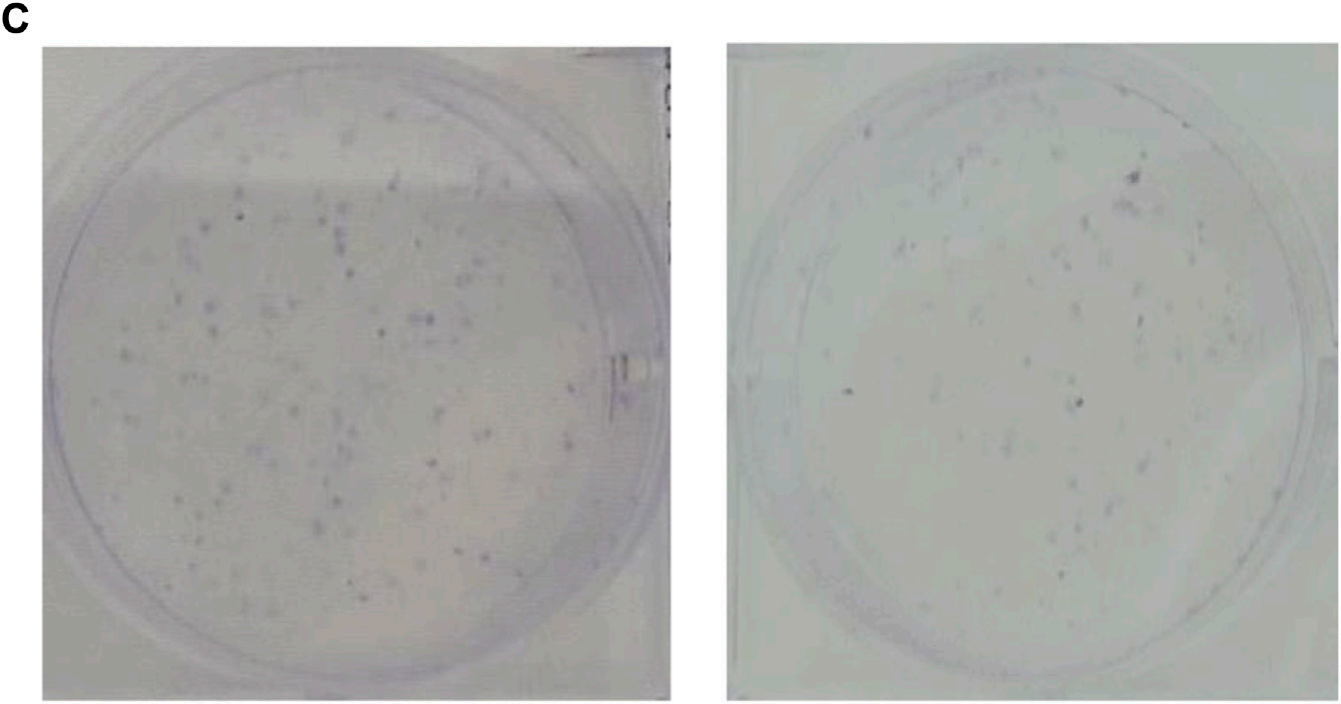
SNHG20 silencing inhibited cell proliferation and cell cycle progression and promoted cell apoptosis in ccRCC cells. 786-O and ACHN cells were transfected with si-SNHG20#1, 2, 3, or si-NC. **(A)** The level of SNHG20 in transfected cells was measured by quantitative real-time PCR. **(B)** The cell viability in transfected cells was assessed via CCK-8 assay. **(C)** Colony formation assays performed with the 786-O and ACHN cells transfected with si-SNHG#1 or si-NC. **(D)** Cell cycle distribution and **(E)** apoptotic status were analyzed in transfected 786-O and ACHN cells using flow cytometry analysis. Statistical significance of differences is indicated as follows: **p < 0.01 and ***p < 0.001.

In the published article, there was an error. In the **Ethics statement** section of page 11 in the article, we mistakenly used “The animal study was reviewed and approved by The study protocol was approved by the Ethics Committee of the First Hospital of Jilin University.”

A correction has been made to **Ethics statement**.

This sentence previously stated:

“The animal study was reviewed and approved by The study protocol was approved by the Ethics Committee of the First Hospital of Jilin University.”

The corrected sentence appears below:

“The clinical sample preparation protocol was reviewed and approved by the Ethics Committee of the First Hospital of Jilin University.”

The authors apologize for these errors and state that this does not change the scientific conclusions of the article in any way. The original article has been updated.

